# The combination of three-dimensional and rotary cell culture system promotes the proliferation and maintains the differentiation potential of rat BMSCs

**DOI:** 10.1038/s41598-017-00087-x

**Published:** 2017-03-15

**Authors:** Yilong Tang, Yan Xu, Zhifeng Xiao, Yannan Zhao, Jing Li, Sufang Han, Lei Chen, Bin Dai, Ling Wang, Bing Chen, Hong Wang

**Affiliations:** 1 Department of Spine Surgery, First Affiliated Hospital of Dalian Medical University, Dalian, 116011 People’s Republic of China; 2 Department of Spine Surgery, Sichuan Provincial Orthopedic Hospital, Chengdu, 610041 People’s Republic of China; 3 Department of Spine Surgery, Dali Bai Autonomous Prefecture People’s Hospital, Dali, 671000 People’s Republic of China; 4 State Key Laboratory of Molecular Developmental Biology, Institute of Genetics and Developmental Biology, Chinese Academy of Sciences, Beijing, 100190 People’s Republic of China; 5 Institute of Combined Injury, State Key Laboratory of Trauma, Burns and Combined Injury, Chongqing Engineering Research Center for Nanomedicine, College of Preventive Medicine, Third Military Medical University, Chongqing, 400038 People’s Republic of China; 6 Department of Biotechnology, College of Life Sciences, Shandong Agricultural University, Taian, 271018 People’s Republic of China; 7 Department of Oncology, First Affiliated Hospital of Dalian Medical University, Dalian, 116011 People’s Republic of China

## Abstract

Bone marrow mesenchymal stem cells (BMSCs) are a good candidate for tissue engineering and clinical application. One of the challenges in its cell therapy is how to quickly obtain an adequate number of seed cells and meanwhile maintain suitable differentiation potential. In this study we combined three-dimensional (3D) collagen porous scaffolds with rotary cell culture system (RCCS) (RCCS-3D) to create a stereoscopic dynamic environment for the amplification of rat BMSCs *in vitro*. The results revealed that this RCCS-3D system could enhance BMSCs’ proliferation and colony formation, as well as maintain the differentiation potential compared with conventional static two-dimensional (2D) and 3D cell culture conditions. In addition, high-throughput microarray analysis showed that gene expressions of RCCS-3D system displayed significant differences in cell proliferation and differentiation compared with static-2D conditions. Thus, RCCS-3D system could provide an effective means for BMSCs cell proliferation *in vitro* and meanwhile maintain differentiation potential in tissue engineering.

## Introduction

Bone marrow mesenchymal stem cells (BMSCs) are ideal seed cells for tissue engineering and regenerative medicine due to their great properties of self-renewal and pluripotency. Moreover, autogenous BMSCs do not have exogenous gene safety issues and ethical problems involved in induced pluripotent stem (iPS) cells^1–3^ and embryonic stem (ES) cells^4^. However, it’s still a challenge in clinical application of tissue-engineering to obtain sufficient quantities of BMSCs through static two-dimensional (2-D) expansion *in vitro* ^5,6^. What is worse, long-term sequential cell passaging results in loss of self-renewal and pluripotency *in vitro* ^7^.

The cells growing space environment and the dynamic environment can influence the seed cell fate^8^. Compared with conventional 2D cell culture, 3D cell culture has received wide attention for it could mimic a native cellular 3D niche *in vivo* to some extent, which is beneficial to the exchanges of nutrition and metabolism, extracellular matrix synthesis and forming of intricate cell-cell and cell-matrix interactions^9^. Extracellular matrix (ECM) plays a critical role in cell proliferation and differentiation in static condition *in vitro* ^10–13^. Collagen, the main component of ECM *in vivo*, is a major component of ECM and comprises the major (80–90%) organic component in mineralized bone tissues^14^. In physiological condition, collagenous stroma is conducive to apatite mineral deposition and the accumulation of noncollagenous and plasma proteins as well as proteoglycans^14–16^. Some studies suggested that 3D microenvironment could maintain the undifferentiated state of stem cells at static status^17–19^. Thus, collagen is a suitable cell carrier for favorable biocompatibility and protein-binding capacity.

Rotary cell culture system (RCCS) cannot only produce rotary movement to simulate microgravity effect by the method of vector-averaged gravity^20,21^ which makes cells losing their intracellular metabolic activity response to gravity and alters the cell perception of gravitational direction, but also provide a dynamic culture system for vital cell movements. Several studies have indicated that the effects of rotary culture on cell fate are multifaceted. Some studies show that rotary culture promoted the proliferation of human adult bone marrow-derived mesenchymal stem cells without scaffolds^22^ and promoted various cell proliferations in rotating liquid vessel^23–25^. Others indicated that rotary culture maintains the undifferentiated state and enhances the neural repair potential of bone marrow stromal cells^26^ and promotes chondrogenesis of human adipose-derived mesenchymal stem cells via the p38 MAPK pathway^27^.

In our study, after two or three passages, isolated BMSCs were seeded in cell culture dishes as static-2D group, on collagen scaffolds placed in dishes as static-3D group and on collagen scaffolds rotating in RCCS as RCCS-3D group, respectively. To investigate the effect of 2D static, 3D static and 3D rotary culture on BMSCs, cells cultured in dishes or scaffolds were directly applied to cell proliferation assay. Cells in each group were expanded for 7 days with proliferating L-DMEM medium and then transplanted into 2D cell culture dishes for evaluating colony-forming ability, osteogenic and adipogenic differentiation potential and the expressions of genes associated with osteogenesis and adipogenesis by quantitative real-time PCR (qPCR). Furthermore, a microarray analysis was introduced to identify potential molecular markers and GO biological process across different culture conditions. We found that RCCS-3D system enhances BMSCs proliferation and maintains the differentiation potential.

## Results

### Characterization of BMSCs

**Figure 1:**
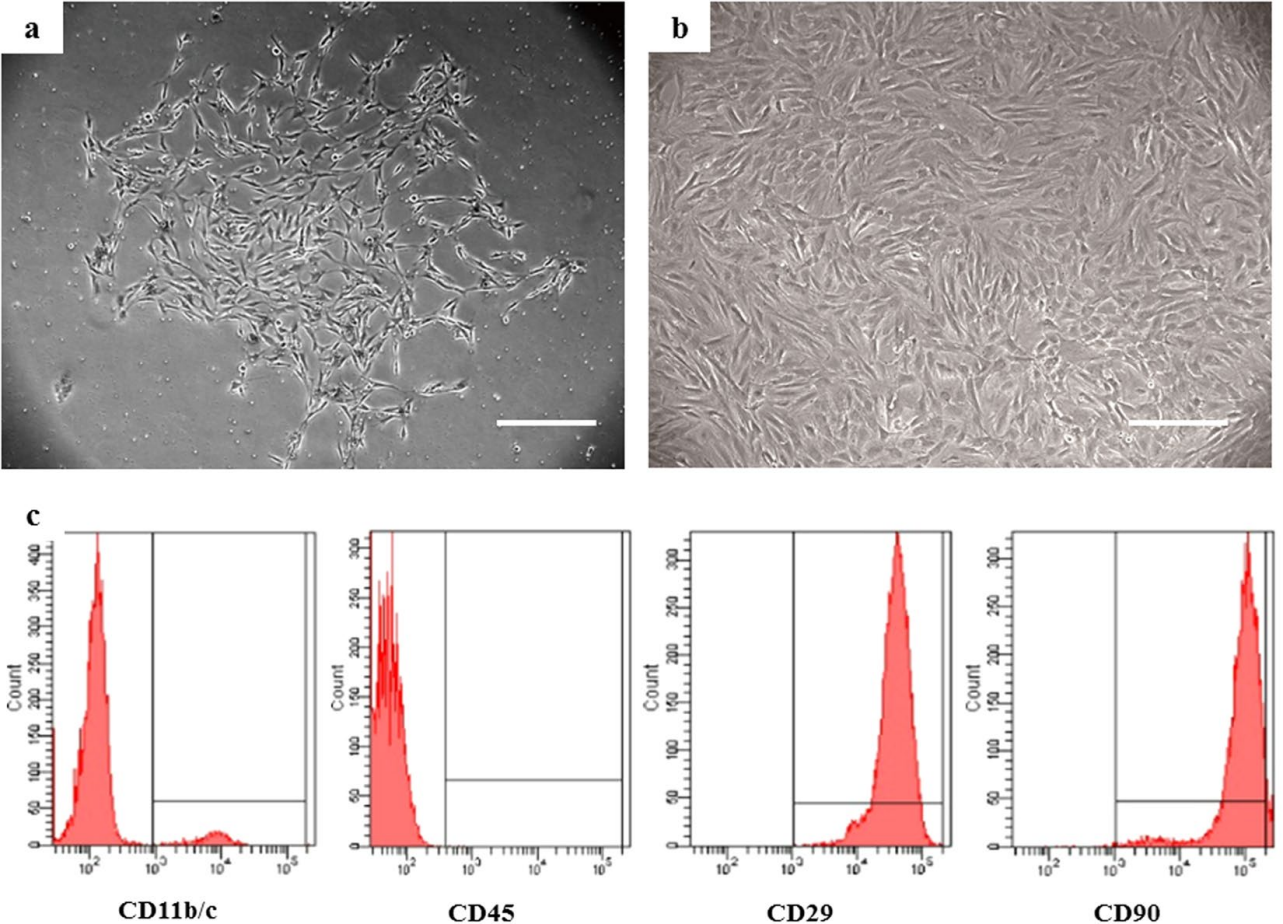
(**a**) Phase-contrast image of BMSCs colonies in the primary culture. Scale bar = 200 μm. (**b**) Phase-contrast image of BMSCs after three passages. Scale bar = 200 μm. (**c**) Fluorescent-activated cell sorting (FACS) analysis revealed that cells were positive for CD29 and CD90 expression and negative for CD11b/c and CD45.

The primary BMSCs displayed a fibroblast-like morphology and developed to visible colonies after 3–5 days culture (Fig. 1a). After 2 weeks, the cells showed their typical big spindle shape morphology and lined almost next to each other (Fig. 1b). To obtain sufficient cell numbers, we performed the follow studies after 2 weeks in culture. Flow cytometry analysis revealed that the cells were uniformly positive for CD29 and CD90, but negative for the hematopoietic surface markers CD11b/c and CD45 (Fig. 1c).

### Properties of collagen scaffolds and establishment of cell culture models

**Figure 2:**
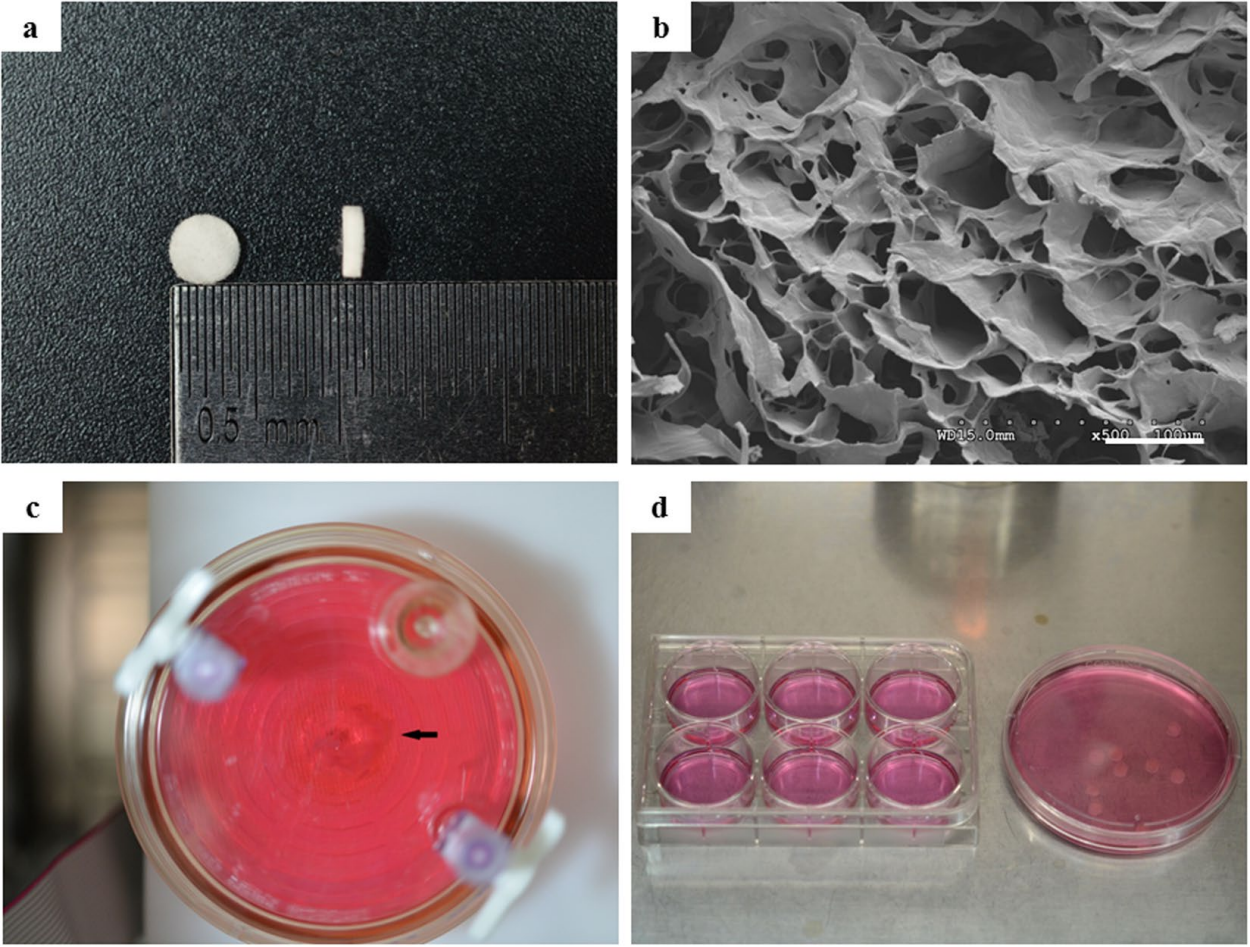
The morphology of BMSCs on 2D culture plate and 3D scaffolds. (**a**) Macrophotograph of 3D collagen porous scaffolds. (**b**) SEM image of 3D porous scaffolds. Scale bar = 400 μm. (**c**) Macrophotograph of BMSCs cultured in RCCS-3D group. (**d**) Macrophotograph of BMSCs cultured in static-2D group and static-3D group.

The collagen material was cut into 4 mm in diameter and 1 mm thick collagen scaffolds (Fig. 2a). SEM analysis showed the collagen scaffolds exhibited interconnecting porous structures whose diameter of pore size reached 40 μm to 100 μm (Fig. 2b). The porous structure of the collagen scaffolds was beneficial to the exchange of nutrition and the metabolism in cultured cells. In RCCS-3D group, cells on collagen scaffolds revolved around the vessel axis at uniform speed in rotating liquid (Fig. 2c). In static-2D and static-3D group, cells were transplanted on dishes and on collagen scaffolds in medium (Fig. 2d).

### Morphology and growth of cells

**Figure 3:**
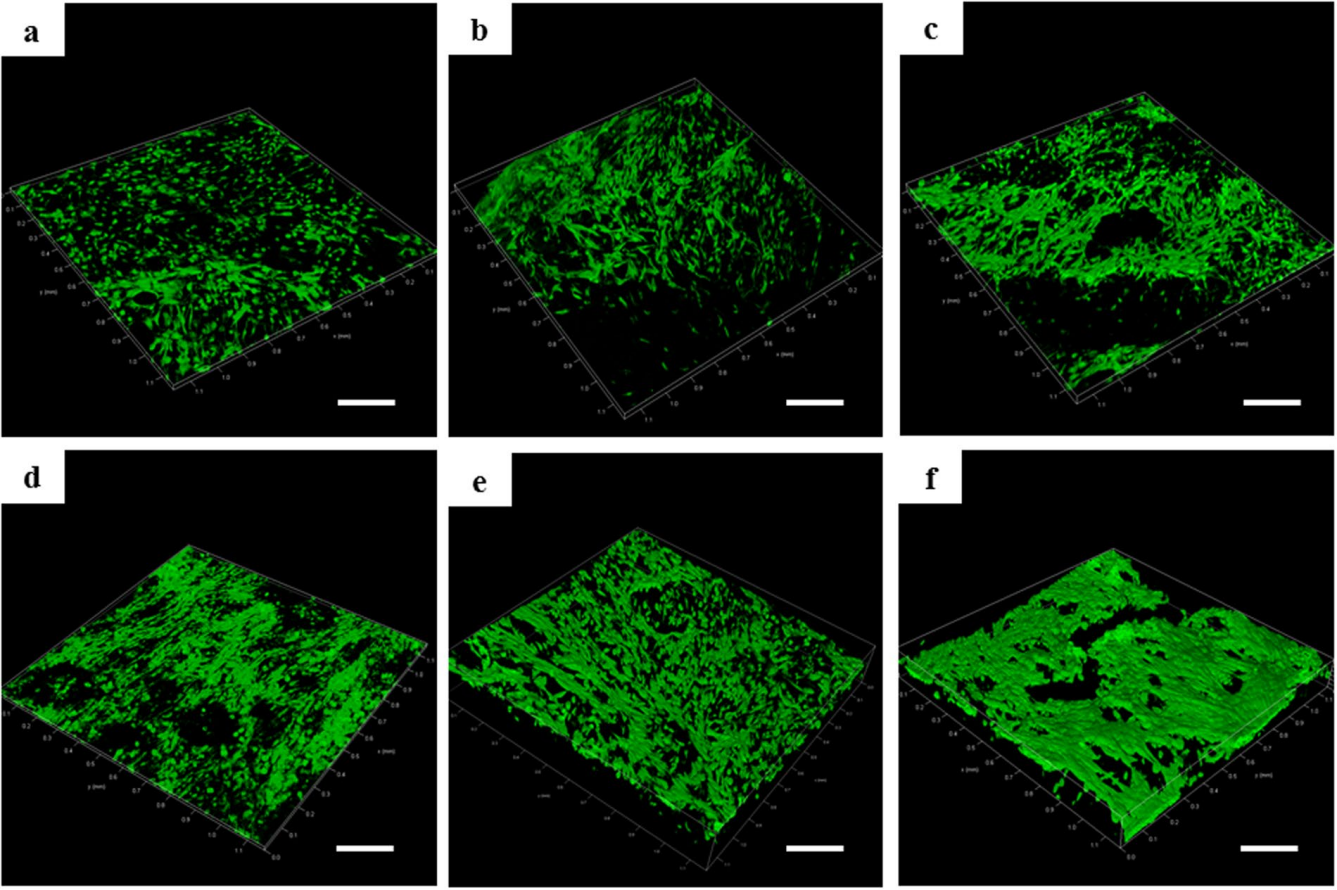
Confocal images of BMSCs stained with FDA in static-2D group after 3 days (**a**), 7 days (**d**), static-3D group after 3 days (**b**), 7 days (**e**), and RCCS-3D group after 3 days (**c**), 7 days (**f**). Scale bar = 200 μm.

**Figure 4:**
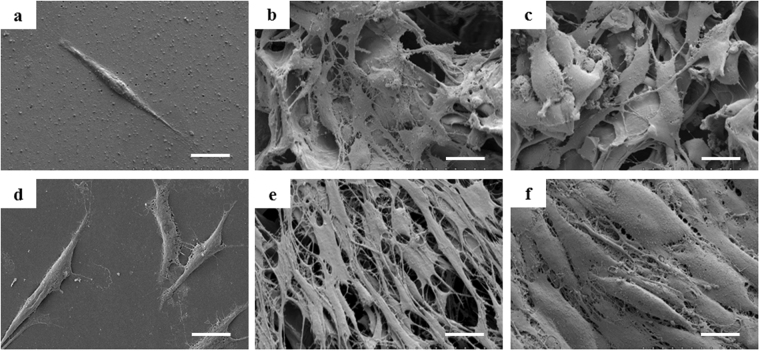
SEM images of BMSCs grown in static-2D group after 3 days (**a**), 7 days (**d**), static-3D group after 3 days (**b**), 7 days (**e**), and RCCS-3D group after 3 days (**c**), 7 days (**f**). Scale bar = 20 μm.

For the analysis of the growing status of the cells in different groups, alive cells were stained by FDA and visualized by laser scanning confocal microscope. At third day, cells in each condition grew well (Fig. 3a,c and e). Cell density in each condition increased with the extension of the cultivation time (Fig. 3b,d and f). However, the cells in RCCS-3D group exhibited a plump morphology expanding along the scaffold structure. Furthermore, it can be seen that cells expanding in RCCS-3D group established tight cell-cell junctions (Fig. 3f). Through SEM analysis, the cells in 2D condition cultured for 3 days showed typical elongated spindle-like morphology adhered to flat plastic surface (Fig. 4a). On the contrary, the static-3D and the RCCS-3D group cells emerged various morphological changes penetrated into the pores of 3D collagen scaffolds (Fig. 4c and e). With the cells expanding to the 7th day, intercellular gaps in the RCCS-3D group almost completely contacted (Fig. 4f), while cells on 2D flat plastic surface proliferated slowly and cells on static collagen scaffolds did not completely form cell-cell tight junctions (Fig. 4b and d).

### Cell proliferation and colony-forming assay

**Figure 5:**
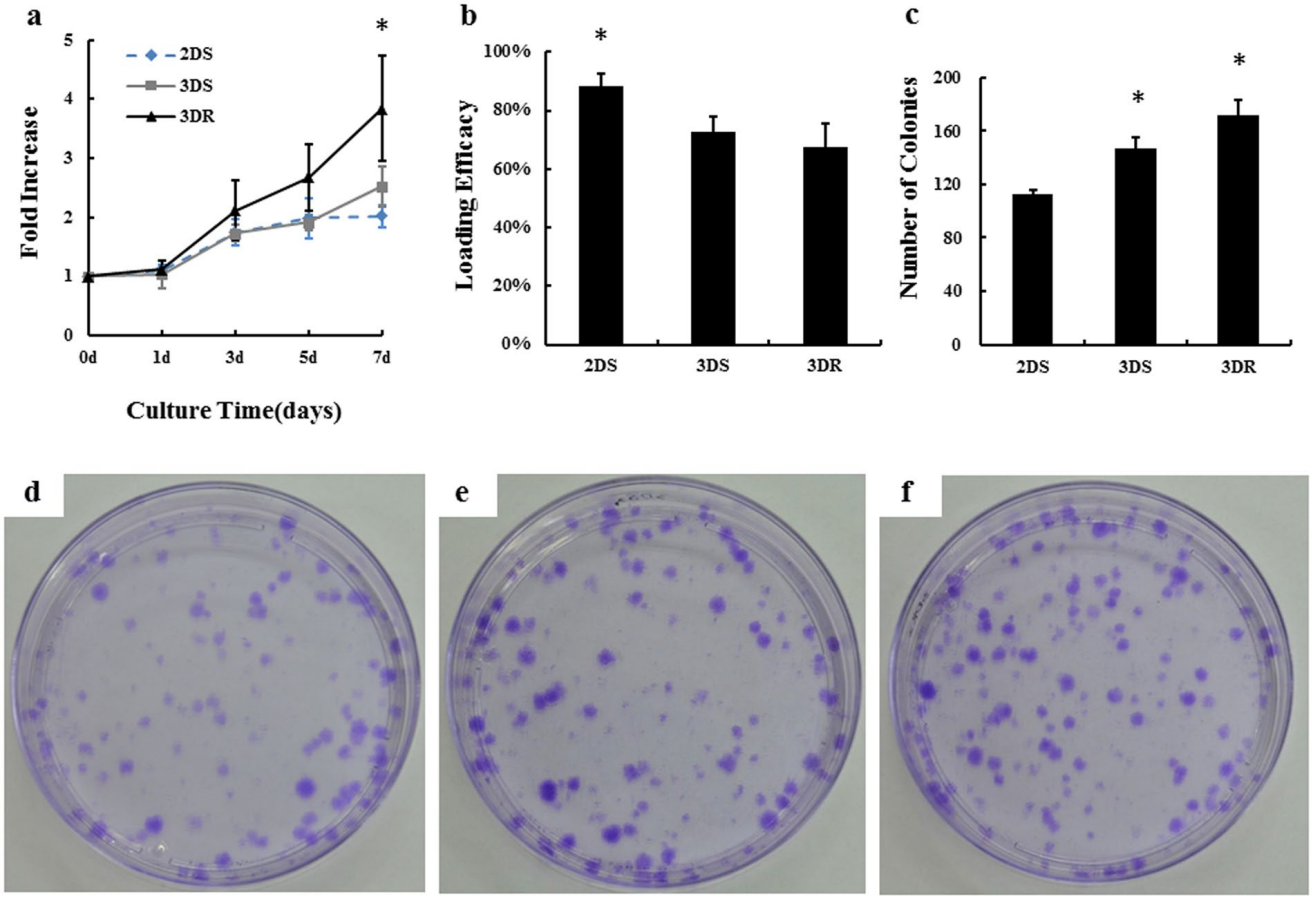
Cell number and CFU analysis of BMSCs cultured in static-2D, static-3D and RCCS-3D groups. (**a**) Proliferation curves of BMSCs in static-2D, static-3D and RCCS-3D groups. The fold increase of cell number was calculated at 0, 1, 3, 5 and 7 day, normalized with the fold increase of cells was set as 0 on the seeding day. The difference on the 7th day was significant by one-way ANOVA analysis (*p < 0.05). (**b**) Loading efficacy of cells in static-2D, static-3D and RCCS-3D groups. (**c**) Statistical analysis of colony numbers of BMSCs in each group (n = 3, *p < 0.05). (**d–f**) Morphology of colonies formed by the cells in static-2D group (**d**), static-3D group (**e**) and RCCS-3D group (**f**) and the colonies were stained with crystal violet.

To investigate the cell growth of BMSCs in 3 groups, we examined cell proliferation at day 0, 1, 3, 5, and 7 by counting the total number of cells. The cells number in each group increased along with the time points (Fig. 5a). The difference between RCCS-3D group and static-2D group or static-3D group on the 7th day was significant (*P* < 0.05). Loading efficacy was designed to evaluate the loading efficacy of cells in each group. We found the loading efficacy of cells on static-2D group, static-3D group and RCCS-3D group were 88.3% ± 4.2%, 72.8% ± 5.2% and 67.0% ± 8.4% (Fig. 5b).

After transferring to 2D 10 mm cell culture dishes and cultured for 12 days, the cells in all the 3 groups formed colonies (Fig. 5d,e and f). However, more colonies were formed in RCCS-3D group than that in static-2D and static-3D conditions (Fig. 5c).

### Microarray data

**Figure 6:**
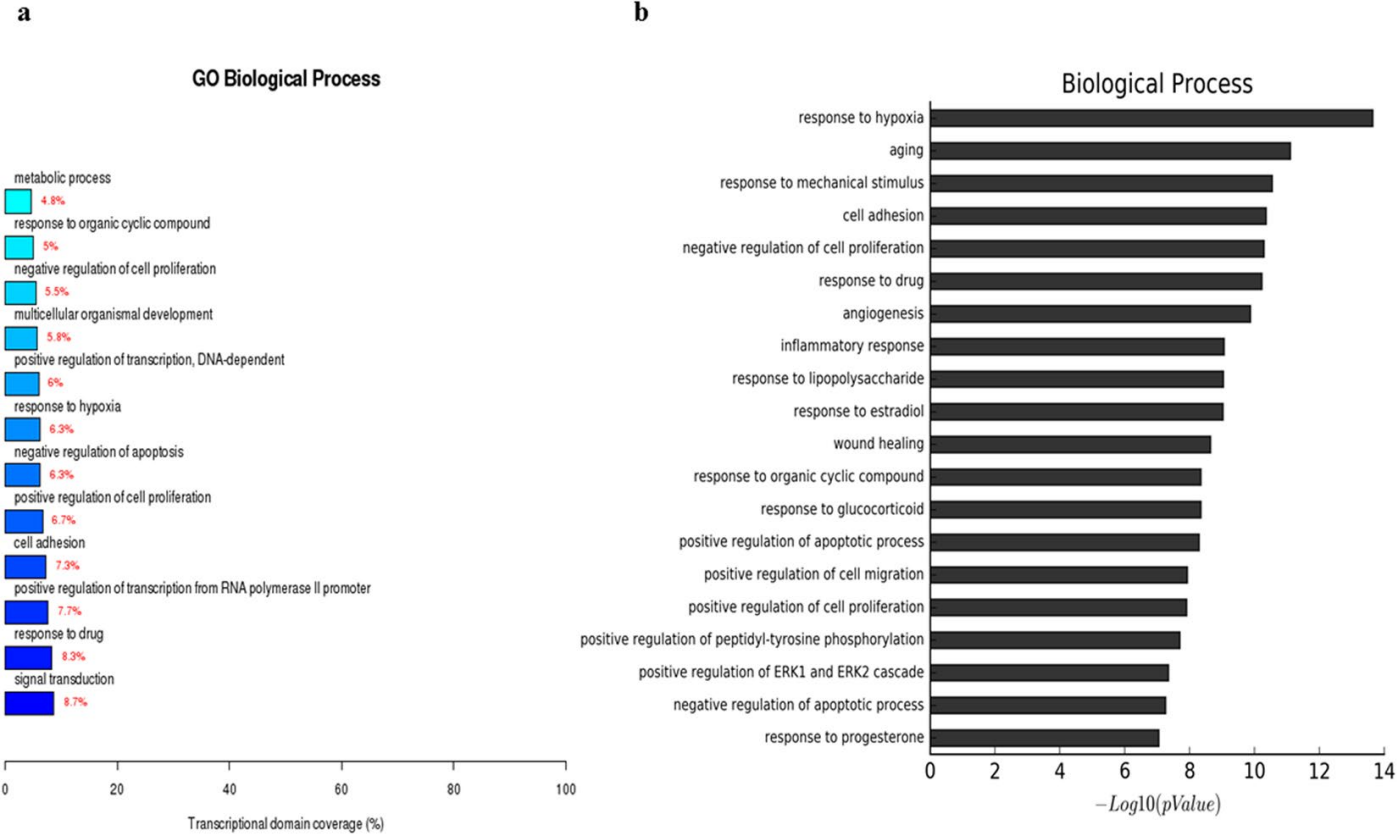
The RCCS-3D vs static-2D group comparison that gene ontology analysis of differentially expressed genes in biological process. (**a**) Top 20 terms based on most significant p value of biological processes. (**b**) Top 12 terms based on the differential genes were most extensive transcriptional domain coverage.

**Figure 7:**
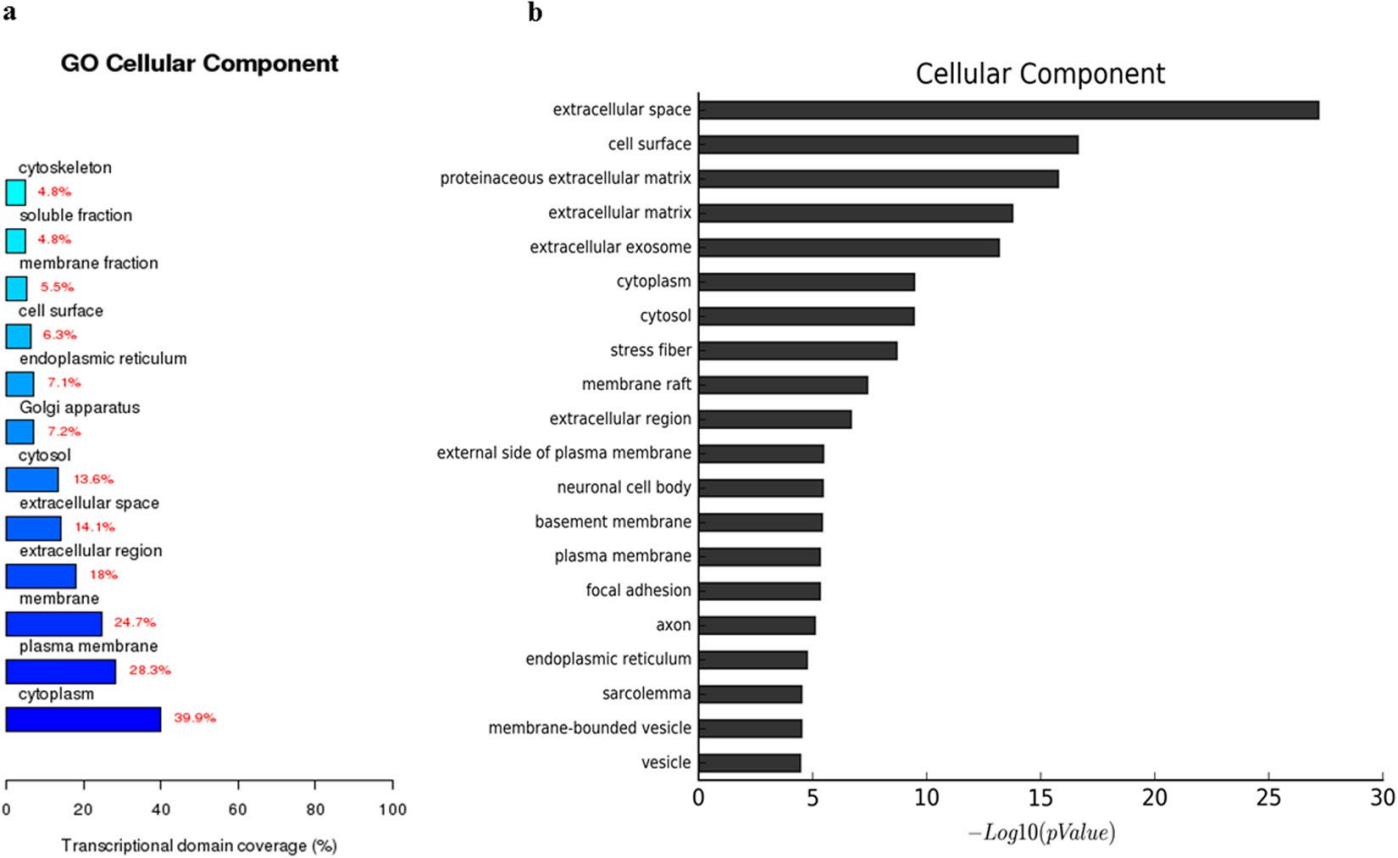
The RCCS-3D vs static-2D group comparison that gene ontology analysis of differentially expressed genes in cellular component. (**a**) Top 20 terms based on most significant p value of biological processes. (**b**) Top 12 terms based on the differential genes were most extensive transcriptional domain coverage.

**Figure 8:**
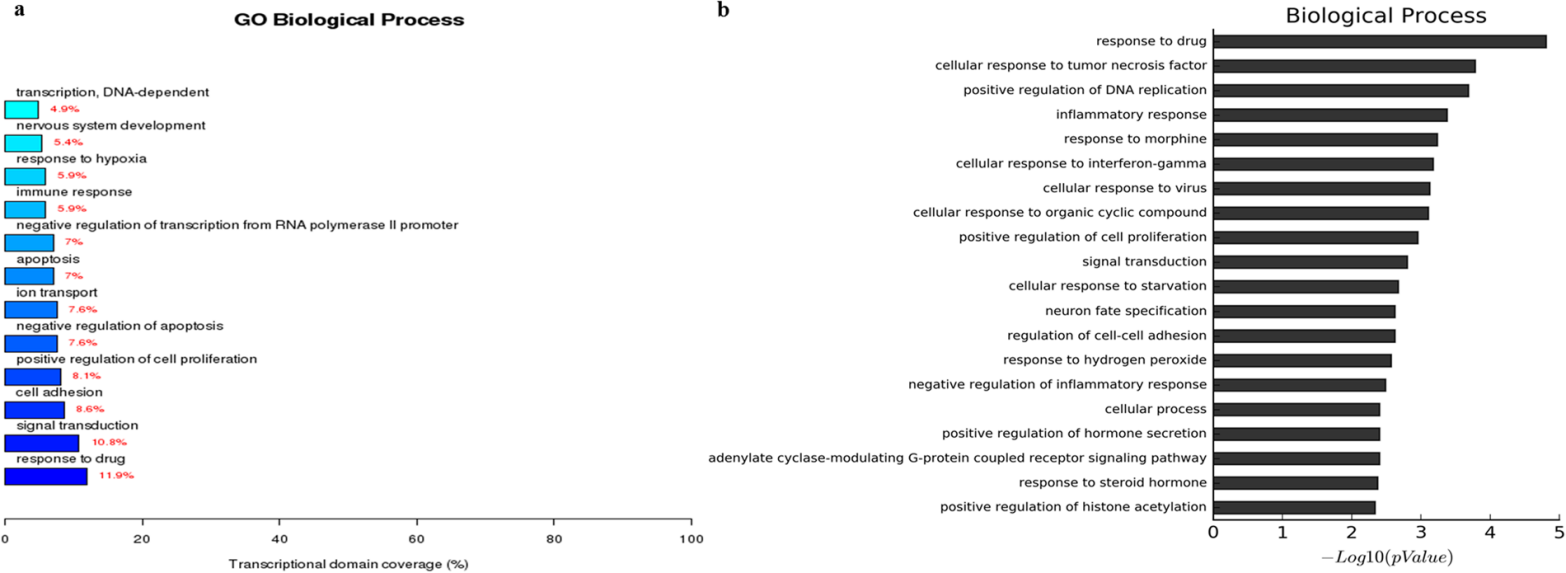
The RCCS-3D vs static-3D group comparison that gene ontology analysis of differentially expressed genes in biological process. (**a**) Top 20 terms based on most significant p value of biological processes. (**b**) Top 12 terms based on the differential genes were most extensive transcriptional domain coverage.

**Figure 9:**
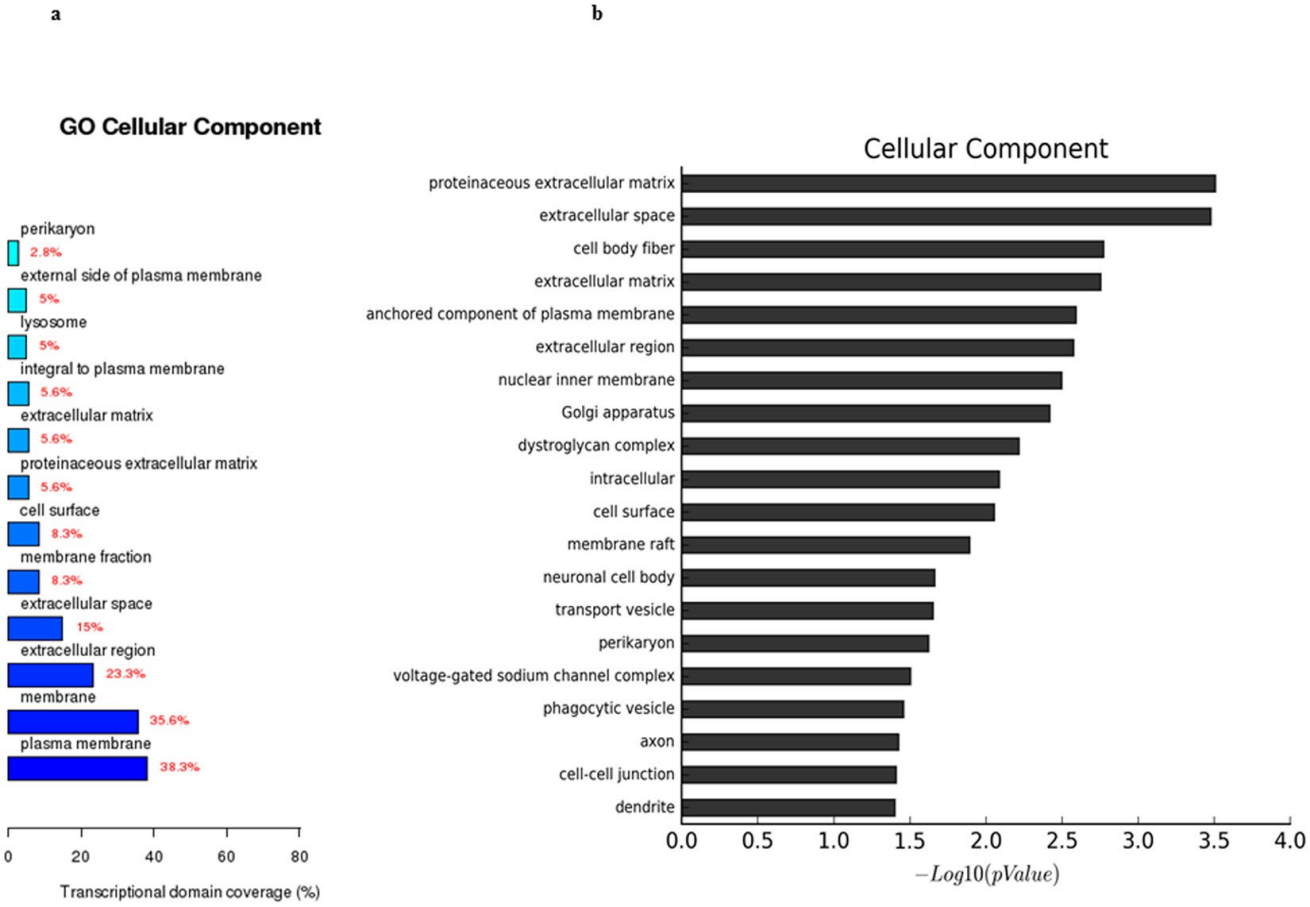
The RCCS-3D vs static-3D group comparison that gene ontology analysis of differentially expressed genes in cellular component. (**a**) Top 20 terms based on most significant p value of biological processes. (**b**) Top 12 terms based on the differential genes were most extensive transcriptional domain coverage.

The gene expression analysis of the three groups showed that 1775 genes were upregulated and 1830 were downregulated in RCCS-3D group compared to that in the static-2D group, 1889 genes were upregulated and 1700 downregulated in static-3D group compared to that in the static-2D group, and 244 genes were upregulated and 465 were downregulated in RCCS-3D group compared to that in the static-3D group. Compared with the transcriptional domain coverage of the differential genes in biological process of static-2D group, RCCS-3D group showed the differences of differential genes mainly concentrated in signal transduction, cell adhesion, cell proliferation, apoptosis, gene transcription, response to hypoxia, multicellular organismal development and metabolic (Fig. 6a). Compared to static-2D group, there were top 20 terms based on most significant p value of biological process in RCCS-3D group were involving in response to hypoxia, cell adhesion, regulation of apoptosis, and regulation of cell proliferation, etc. (Fig. 6b). And we can also see the results of the terms based on most significant p value in cellular component differential genes analysis and the transcriptional domain coverage of the differential genes analysis were mainly involving in focal adhesion, intracellular organelles and extracellular matrix, etc. (Fig. 7a,b). In addition, results of the terms based on biological process and cellular component analysis in RCCS-3D group vs static-3D group were less significantly differential compared with RCCS-3D group vs static-2D group (Figs 8a,b and 9a,b). The top 20 up-regulation gene expression of positive regulation of cell proliferation analysis and negative regulation of apoptotic process in biological process analysis were displayed in Tables 1 and 2.

**Table 1:** Top 20 up-regulation gene expression of positive regulation of cell proliferation in biological process analysis.

UniGene ID	Gene Title	Gene Symbol	Regulation	Fold Change (3DR vs 2DS)
Rn.53973	colony stimulating factor 3 (granulocyte)	Csf3	up	171.62158
Rn.33146	cathelicidin antimicrobial peptide	Camp	up	48.08884
Rn.102416	secreted frizzled-related protein 2	Sfrp2	up	40.572308
Rn.198483	interleukin 11	Il11	up	37.9506
Rn.10232	adrenomedullin	Adm	up	30.882835
Rn.6282	insulin-like growth factor 1	Igf1	up	21.025723
Rn.42897	epiregulin	Ereg	up	18.446724
Rn.98842	fibroblast growth factor 7	Fgf7	up	15.823176
Rn.44285	colony stimulating factor 2	Csf2	up	11.562983
Rn.9873	interleukin 6	Il6	up	10.630553
Rn.10468	hepatocyte growth factor	Hgf	up	9.064637
Rn.48749	wingless-type MMTV integration site family, member 5A	Wnt5a	up	7.5712037
Rn.130193	cardiotrophin-like cytokine factor 1	Clcf1	up	7.039917
Rn.44216	KIT ligand	Kitlg	up	6.747355
Rn.1716	interleukin 6 receptor	Il6r	up	5.9479833
Rn.162144	T-box 3	Tbx3	up	5.3641796
Rn.9664	activating transcription factor 3	Atf3	up	5.3632503
Rn.10568	amphiregulin	Areg	up	4.8201013
Rn.1923	vascular endothelial growth factor A	Vegfa	up	4.6613054
Rn.12138	interleukin 6 signal transducer	Il6st	up	4.5940213

**Table 2:** Top 20 up-regulation gene expression of negative regulation of apoptotic process in biological process analysis.

UniGene ID	Gene Title	Gene Symbol	Regulation	Fold Change (3DR vs 2DS)
Rn.119611	angiopoietin-like 4	Angptl4	up	679.1727
Rn.16279	twist homolog 2 (Drosophila)	Twist2	up	159.442
Rn.11412	endothelin receptor type B	Ednrb	up	111.2116
Rn.161953	angiopoietin 1	Angpt1	up	84.16098
Rn.198135	B-cell CLL/lymphoma 3	Bcl3	up	55.382526
Rn.102416	secreted frizzled-related protein 2	Sfrp2	up	40.572308
Rn.10488	superoxide dismutase 2, mitochondrial	Sod2	up	21.491787
Rn.19770	BCL2-related protein A1d	Bcl2a1d	up	16.618256
Rn.107266	chemokine (C-X3-C motif) ligand 1	Cx3cl1	up	14.690553
Rn.6800	platelet factor 4	Pf4	up	14.353208
Rn.31427	microphthalmia-associated transcription factor	Mitf	up	11.888482
Rn.44285	colony stimulating factor 2 (granulocyte-macrophage)	Csf2	up	11.562983
Rn.9873	interleukin 6	Il6	up	10.630553
Rn.1780	clusterin	Clu	up	10.37447
Rn.10468	hepatocyte growth factor	Hgf	up	9.064637
Rn.48749	wingless-type MMTV integration site family, member 5A	Wnt5a	up	7.5712037
Rn.27923	BTG family, member 2	Btg2	up	7.554541
Rn.6051	dipeptidase 1 (renal)	Dpep1	up	7.429901
Rn.44216	KIT ligand	Kitlg	up	6.747355
Rn.62694	nuclear receptor subfamily 4, group A, member 3	Nr4a3	up	5.482495

### Adipogenic differentiation ability analysis

**Figure 10:**
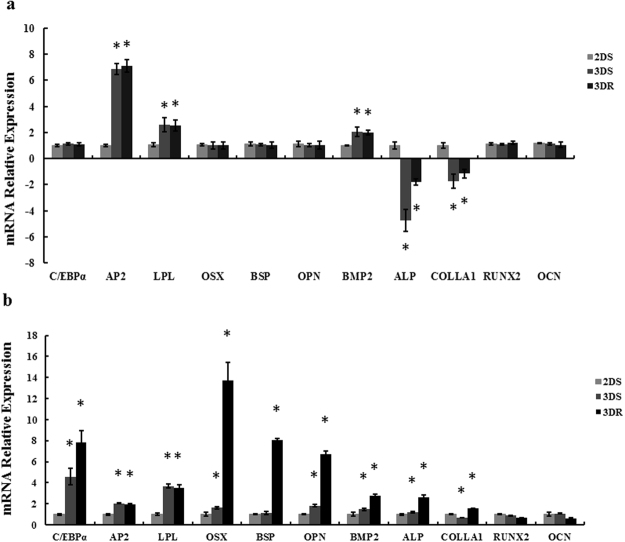
Adipogenic and osteogenic differentiation of BMSCs in static-2D, static-3D and RCCS-3D groups. (**a**) Q-PCR analysis of adipogenic gene expression levels and osteogenic gene expression levels before BMSCs in each group transplanted to 2D plates for 21 days adipogenic and osteogenic differentiation (N = 3, *p < 0.05). (**b**) Q-PCR analysis of adipogenic gene expression levels and osteogenic gene expression levels after BMSCs transplanted to 2D plates for 21 days adipogenic and osteogenic differentiation of BMSCs in each group (N = 3, *p < 0.05).

**Figure 11:**
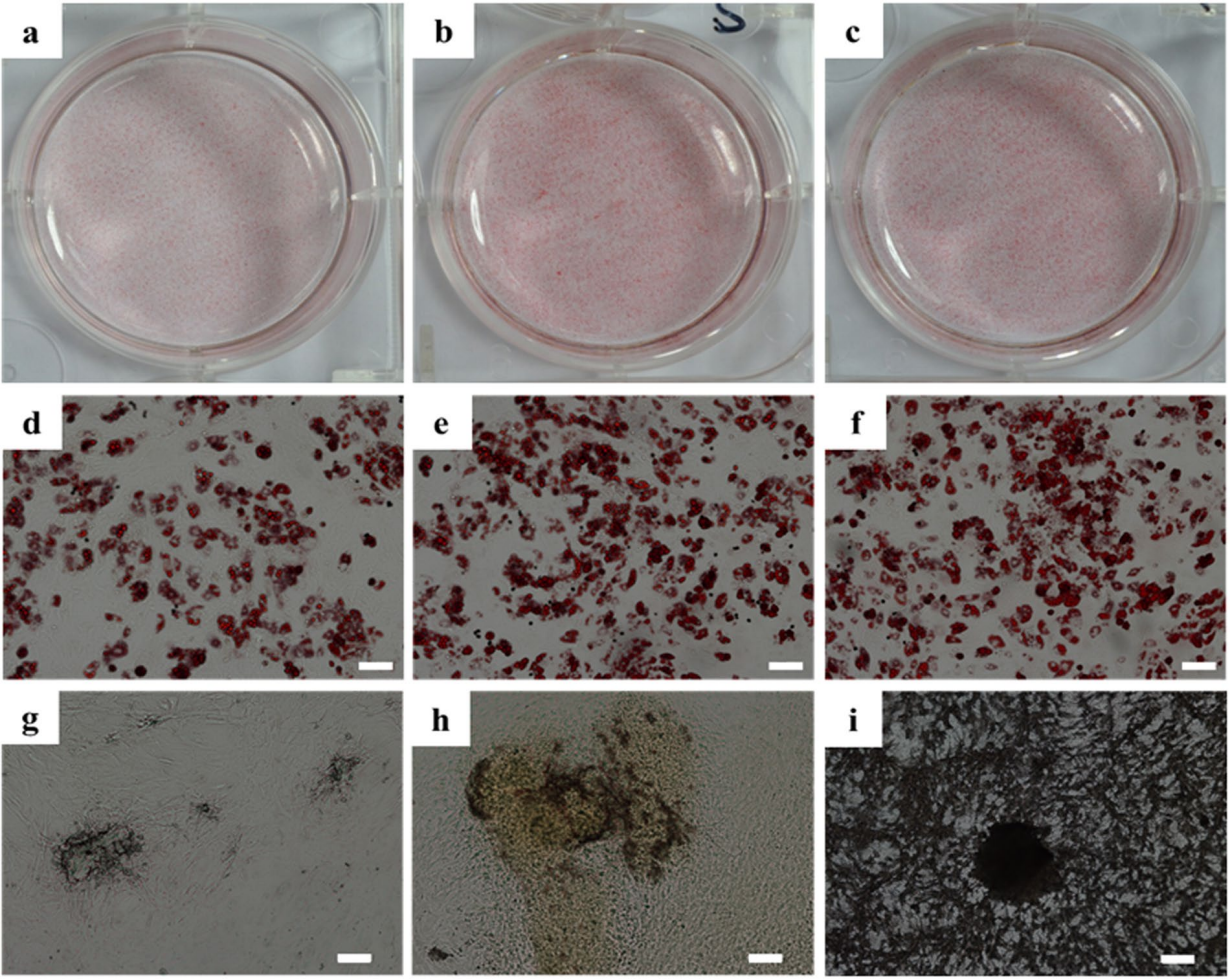
Oil red O staining (**a–f**) and Von kossa staining (**g–i**) of BMSCs transplanted to 2D plates for 21 days adipogenic and osteogenic differentiation.

To detect the adipogenic differentiation of the cells in different culture conditions after 7 days expanding, each group cells were transplanted to 2D plates and induced to adipogenic differentiation for 21 days. Before transplanted to 2D plates and induced to adipogenic differentiation, the RNA transcripton level of the markers of adipocytes such as adipocyte fatty acid binding protein(AP2) and Lipoprotein lipase (LPL) of the static-3D and RCCS-3D groups were increased compared to that of the static-2D group (Fig. 10a). And the RNA transcripton level of the markers of adipocytes such as CCAAT/enhancer binding protein α(C/EBPα), adipocyte fatty acid binding protein(AP2) and Lipoprotein lipase (LPL) of the static-3D and RCCS-3D groups were increased significantly compared to that of the static-2D group (Fig. 10b). Meanwhile, the adipogenic differentiation images of the three groups suggested that more red lipid droplets stained by oil red O were formed in static-3D (Fig. 11b and e) and RCCS-3D groups (Fig. 11c and f) than that in the static-2D group (Fig. 11a and d).

### Osteogenic differentiation ability analysis

To detect the osteogenic differentiation of the cells in different expanding conditions after 7 days, each group cells were transplanted to 2D plates and induced to on for 21 days. Before transplanted to 2D plates and induced to osteogenic differentiation, the RNA transcription level of the markers of osteocytes such as bone morphogenetic protein 2(BMP2), Alkaline phosphatase (ALP) and Collagen type I (ColI) of the static-3D and RCCS-3D groups were increased compared to that of the static-2D group (Fig. 10a). And the expression levels of osteogenesis-related genes, odontogenesis-related transcription factors Osterix (OSX), bone sialoprotein (BSP), osteopontin (OPN), bone morphogenetic protein 2(BMP2), Alkaline phosphatase (ALP), osteocalcin(Ocn), runt related transcription factor 2 (Runx2) and Collagen type I (ColI) were significantly elevated (P < 0.05) in RCCS-3D group compared to that in the static-2D group and static-3D group at day 21 when normalized to GAPDH (Fig. 10b). And the Von Kossa staining was carried out to visualize the calcium deposition in cells. Cells derived from RCCS-3D group (Fig. 11g) tended to form more calcium deposition stained by silver compared to that in the static-2D group (Fig. 11h) and static-3D group (Fig. 11i).

## Discussion

In this study, we combined three-dimensional collagen porous scaffolds with rotary cell culture system to pursue an available approach to expand sufficient seed cells and maintain their pluripotency for tissue engineering applications. 3D porous natural collagen scaffolds were used to provide an appropriate 3D niche for accreting ECM due to their biocompatibility and protein-binding capacity, which was conducive to MSCs to maintain the differentiation potential. In the 3D microenvironment, the cell populations could interface with their surrounding environment and expand with similar to that occurred during *in vivo*. In addition, the results of cell number count and CFU assay showed RCCS could promote the cell proliferation and self-renew of BMSCs on the 3D collagen scaffold. To some extent, 3D rotary cell culture may be a feasible method to massively amplify seed cells for bioengineering *in vitro*.

Self-renewal is the intrinsic property of stem cells that allows them to extensively proliferate, prevent apoptosis, and avoid loss of multiple differentiation potential. It is well-known that mesenchymal stromal cells cannot avoid aging and loss of their proliferative capacity and differentiation potential progressively in traditional static-2D culture system. The 3D structure and dynamic environment of RCCS-scaffold system provides suitable conditions for deposition of extracellular matrix. Extracellular matrix has a positive role on the cell proliferation, anti-apoptosis resistance, anti-aging and cell differentiation^28–30^. RCCS-3D system has promoted the cell proliferation compared with static-2D system based on the cell fold increasing curves and colony-forming assay. Moreover, the porous 3D scaffold structure and liquid surface tension of cell suspension may cause a certain amount of cell loss on the cell culture dishes during cell inoculation. Therefore, the actual number of 3D scaffolds cells ought to be less than the count number, that is, it may be systematic error that the actual fold increase values of 3D groups may be higher than the ones of experimental data. And perhaps this is why the loading efficient of 3D groups were lower than static-2D group. Additionally, the cell bodies of RCCS-3D group displayed more plump and full morphology. And RCCS-3D system might promote the production of intracellular and extracellular based on the cellular component analysis of microarray data, which may indicate that the system enhanced cell biological activity. According to the results of microarray analysis, the differential genes of RCCS-3D group showed significant differences in biological process including response to hypoxia, aging, regulation of apoptosis, proliferation and cell migration compared with static-2D group. Moreover, compared with the transcriptional domain coverage of the differential genes in static-2D group, RCCS-3D group showed the differences of differential genes mainly concentrated in signal transduction, cell adhesion, cell proliferation, apoptosis, gene transcription, response to hypoxia, multicellular organismal development and metabolic. Therefore, it is easy to see that RCCS-3D cell culture system showed significant differences in cytoplasm and their organelles as well as the extracellular matrix compared with traditional static-2D cell culture.

According to the result of the regulation of proliferation and apoptosis in microarray analysis, we can learn some enlightenments that RCCS-3D system enhanced the expression of genes related to proliferation and apoptosis. Angiopoietin-like 4 is a potential anti-apoptotic factor for MSCs. Previous studies have reported that Angptl4 protects MSCs from hypoxia/SD-induced apoptosis by increasing the Bcl-2/Bax ratio and the MMP^31^. Other studies also reported that Angpt1 enhanced the survival and functional recovery of transplant MSC via PI3K/Akt pathway activation^32^. Twist can block the myogenic differentiation, leading to halting the terminal differentiation and inhibition of the apoptosis^33^. SFRP2, an important autocrine factor for MSCs themselves, has recently been highlighted for its therapeutic potential in tissue engineering. Recent studies have shown that sFRP2-expressing MSCs exhibit an increased proliferation rate and inhibit both MSC apoptosis and their differentiation along osteogenic and chondrogenic lineages by blocking the effect of canonical Wnt3a and inhibiting both Wnt and bone morphogenic protein (BMP) signaling pathways^34,35^. Epiregulin (Ereg), one member of the EGF family, can activate MEK/Erk and JNK signalling pathways. Moreover, a previous study has demonstrated that blocking JNK signalling could abolish the effect of Ereg on the proliferation of human dental stem cells, suggesting that JNK signalling was also involved in EREG-mediated cell proliferation^36^. Recently, Platelet factor 4 (PF4) has been suggested to enhance the ability of adherent and proliferation of BMSC while inhibiting its apoptosis^37^. Insulin-like growth factor 1 (IGF1) is thought to be a growth factor found in the fast-growing stem cell clones to induce BMSCs proliferation^38^. Additionally, a study shows that IL-6 is a critical factor for maintaining the stemness of adult human bone marrow-derived MSCs^39^. It regulates both MSC proliferation and inhibition of differentiation through ERK1/2 activation as the key pathway.

Microenvironment and mechanical stimulation of ECM have great impact on cellular activities^40^. The space structure of scaffold provides complex surfaces for cell attachment and growth. In Fig. 4, we can see that multiple contacts of cells and 3D interface in scaffold displayed cell morphology diversity that are analogous to axon and soma of neurons differing from typical spindle cell morphology in static-2D system. According to biological process and cellular component of microarray analysis, RCCS-3D system vs static-2D system showed significant differences in cell adhesion, extracellular matrix microenvironment and response to mechanical stimulation. Focal adhesions provide cells mechanical transmission pathways to respond to their microenvironment^41^. ECM elasticity is able to regulate FAK activity to trigger relative signal molecules to regulate cell differentiation and different elastic substrate can lead to different results^42^. The potential impact of prior expansion of the rat BMSCs in plastic dishes on the differentiation potential of the post-expansion on 3D scaffolds could not be avoided in our study for the rare number and the separation of original MSCs. The impact might have always existed during the prior expansion, which could also influence subsequent differentiation. The rotating mechanical environment and the multipath contact conduction of 3D space structure in scaffold provide complex force field environment for cells to sense the constantly changing direction of gravity, which is similar to different matrix elastic strength mechanical stimulation. The cells in RCCS-3D system may exhibit multicellular organismal differentiation potential.

In this study, after the 7 days expanding in proliferation culture medium, the cells in each group were transplanted to 2D plates and induced to adipogenic and osteogenic differentiation. And we assayed mRNA expressions of the essential adipogenesis-related and osteogenesis-related markers. The results exhibited that more red lipid droplets stained by oil red O in static-3D and RCCS-3D groups in comparison with that in static-2D group and more calcium deposition stained by silver compared to static-2D group and static-3D group. The expressions of both adipogenic and osteogenic genes were upregulated significantly in varying degrees during adipogenic and ostogenic differentiation. C/EBPa is an essential transcription factor for the induction of terminal adipocyte differentiation^43^. Fatty acid binding protein 4/AP2 (Fabp4), a member of the cytoplasmic fatty acid-binding protein family, was detected to express in adipose tissue and its expression was highly regulated during the differentiation of adipocytes^44^. LPL is synthesized in tissues involved in fatty acid metabolism^45^. A study demonstrated that *ex vivo* expansion of MSCs on the collagen matrix results in the retention of the adipogenic differentiation potential *in vitro*. Moreover, the transcript levels of Fabp4 and LPL were demonstrated to be significantly higher in the cells *ex vivo* expanded on the collagen matrix in comparison with the cells *ex vivo* expanded on cultured on TCP^46^. Recent study has identified that JNK-dependent noncanonical WNT-5a signaling is important to maintain the potential of multipotent stem cells to undergo osteogenesis^47^. It is possible that the culture method in our study involving the dynamic and 3D tissue-engineering model stimulates the up-regulation of wnt5a (Table 2), suggesting that this culture system is beneficial for maintaining the multiple differentiation potential of the adult stem cells for a long term growth *in vitro* ^48^. Osterix and Runx2 are two essential transcription factors for osteoblast differentiation and bone formation^49^. OPN is highly expressed during the last stage of bone formation, namely the mineralization period^50^. ALP is an earlier osteogenic marker that increases rapidly with the proliferation and external secretion of osteoblasts, while BSP has activated the function in the initiation of mineralization, both of which are relatively mature osteogenic differentiation markers^51^. The increased oil red staining and Von Kossa staining in RCCS-3D group as well as the assays of mRNA expressions of adipogenesis-related and osteogenesis-related markers suggested that RCCS-3D system could maintain the potential of adipogenic and osteogenic differentiation when MSCs loading on the collagen 3D scaffolds after a long-term culture, while 2D BMSCs tended to lose their differentiation potential after expanding for same long- term time.

In summary, we developed an effective RCCS-3D means for BMSCs cell proliferation *in vitro* and meanwhile maintain differentiation potential in tissue engineering *in vitro*. The mechanisms of 3D dynamic BMSCs culture are complicated and multiple signal pathways including growth, differentiation, and apoptosis are involved. Further studies on the internal connection among the up-regulated genes should shed light on the mechanism by which the combination of three-dimensional culture and RCCS promotes the proliferation and maintains the differentiation potential of BMSCs.

## Methods

### Collagen scaffold preparation

The collagen material was provided by Zhenghai Biotechnology Inc. (Shandong, China). The preparation method of collagen was described previously^52^. Briefly, collagen material was cut into 4 mm in diameter and 1 mm high cylinder. Then, the collagen scaffolds were crosslinked according to the procedure adopted from Zhao *et al.* ^53^. Briefly, the scaffolds were soaked into 2-morpholinoethanesulfonic acid solution (pH 5.6) with Nhydroxysulfosuccinimide (NHS, 1.2 mg/mL) and 1-ethyl-3-(3-dimethyl aminopropyl) carbodiimide (EDC, 3 mg/mL, respectively). After crosslinking at 37 °C for 4 hours, the scaffolds were washed with 0.1 M Na_2_HPO_4_ for 4 hours and with 4 M NaCl overnight to remove the remaining crosslinking reagents, followed by washing with ddH_2_O for half an hour at least eight times, and then freeze-dried overnight. At last, the collagen scaffolds selected based on the previous size standard at 4 mm (diameter) × 1 mm (height) were sterilized by gamma irradiation before further experiments.

### Preparation of rBMSCs and cell-scaffold complexes

Bone marrow mesenchymal stem cells (BMSCs) were isolated from the marrow cavities of tibias and femora of Sprague Dawley rat (8 weeks). The animal experimentation was performed in accordance with NIH guidelines for the care and use of laboratory animals (The study was conducted in accordance with the Declaration of Helsinki and with the Guide for Care and Use of Laboratory Animals as adopted and promulgated by the United National Institutes of Health. All experimental protocols were approved by the Review Committee for the Use of Human or Animal Subjects of Dalian Medical University). Briefly, the marrow was flushed out from the tibias and femora with Low Glucose Dulbecco’s Modified Eagle’s Medium (L-DMEM) containing 100 U/mL penicillin and 100 mg/mL streptomycin. Cells were centrifuged and suspended in proliferating L-DMEM medium containing 10% fetal bovine serum (FBS), 100 mmol/L non-essential amino acids, 100 mmol/L sodium pyruvate, 2 mmol/L L-glutamine, 100 U/mL penicillin, and 100 mg/mL streptomycin. Then they were placed on a culture dish and cultured in an incubator under humidified atmosphere of 5% CO_2_ in air at 37 °C. Nonadherent cells were removed on day 3 and the proliferating medium was changed every 2 days. Cells were passaged at 80% confluence. After two or three passages, cells were used in following tests. Collagen scaffolds were placed in a 100 mm culture dish, 10 ul of 2 × 10^4^ cells suspension was dropwise added on the surface of the collagen scaffold, and the cell-scaffold complex was incubated in 37 °C, 5% CO_2_ incubator for 4 hours. After 4 hours, the cell-scaffold complexes of RCCS-3D group were removed in a sterile container rotating culture system, and 50 ml cell culture medium was added slowly until filling with a 10 ml syringe vent emptying bubble in the rotating culture system container. After the container is mounted to the rotary cultivator machine bed, adjusting a rotational speed of 20 rev/min. The system is placed in the rotary cultivator 37 °C, 5% CO_2_ incubator. The cell-scaffold complexes of static-3D group were removed in cell culture dishes added with 50 ml cell culture medium. The cells of static-2D group were seeded directly in cell culture dishes.

### Flow cytometry (FCM) analysis

The third passage MSCs were harvested by digested with 0.25% trypsin, and suspended in PBS containing 2% FBS. Cells were incubated on ice for 30 min with anti-rat antibodies (BioLegend) conjugated with fluorescein isothiocyanate (FITC) or phycoerythrin (PE). Markers of BMSC (CD29-PE and CD90-PE) and Hematopoietic cell (CD11b/c-FITC and CD45-PE) were evaluated and analyzed respectively. FITC- or PE-conjugated IgG were used as isotype control. After washing three times, cells were analyzed on Fluorescence Activated Cell Sorter (FACS).

### Fluorescein diacetate(FDA) for living cell staining

The samples of the three groups were washed with PBS and incubated in 10 μg/ml fluorescein diacetate(FDA) for 30 seconds at room temperature. Then the scaffolds were washed at least three times with PBS and observed with laser scanning confocal microscope (Leica).

### Scanning electron microscopy (SEM) analysis

The surface morphological characteristics of scaffolds and the cell morphology of static-2D group, static-3D group and RCCS-3D group cells were observed with SEM respectively. After the cells in each group were cultured for 3 days and 6 days, they were gently removed from medium and fixed in 4% glutaraldehyde at 4 °C overnight. Subsequently, the samples were dehydrated by a gradient concentration ethanol series of 30%, 50%, 75%, 80%, 85%, 90%, 95% and 100% and then immersed in isoamyl acetate overnight. After CO_2_ critical point drying, the samples were coated with gold and analyzed by a scanning electron microscopy (Hitachi Science Systems).

### Cell proliferation assay

Cell proliferation assay was performed with cell counting. BMSCs were seeded with an initial density of 20,000 cells per scaffold or 8000 cells per 35 mm culture dish. Every sample was cultured in proliferating L-DMEM medium and the medium was changed every 3 days. After the cells in each group were cultured for 0, 1, 3, 5 and 7 days, they were digested with 0.25% trypsin to count the cell number respectively. Cell multiplications were expressed as a relative value that the fold increase of cells was set compared with the seeding cell density. The time of seeding after 4 hours was set as 0 day.

### Colony-forming unit-fibroblasts (CFU-F) assay

Cells cultured for 7 days in each group were harvested by treatment with 0.25% trypsin. 1000 cells were plated in 10 cm culture dishes and three replicate plates were used for each group. Then the cells were cultured for 12 days and the medium was changed every 3 days. Upon harvesting, the cells were washed with PBS and stained with 0.25% crystal violet solution for 10 min at room temperature. After washing with PBS, the colonies (containing > 50 cells) were counted with microscopic observation.

### RNA isolation and quantitative real-time PCR

Total RNA was extracted from the cultured cells with Trizol (Invitrogen) reagent, and the contaminated genomic DNA was removed with Deoxyribonuclease I (Invitrogen). The first-strand complementary cDNA was synthesized using the SuperScriptTM III Reverse Transcriptase (Invitrogen). qPCR was performed using Power SYBR Green PCR Master Mix (Applied Biosystems) in combination with a CFX96 system (Bio-Rad). The relative mRNA level was expressed as fold change relative to untreated controls after normalization to the expression of GAPDH by the 2^−ΔΔCT^ method. The thermocycler parameters were 95 °C for 10 min, followed by 40 cycles of 95 °C for 15 s and 60 °C for 1 min. The target genes and primer sequences are listed in Table 3.

**Table 3:** Primer sequences used for qPCR amplification.

Gene Title	Gene Symbol	primer sequence
Glyceraldehyde-3-phosphate dehydrogenase	GAPDH	F:5¡^−^-AGAGACAGCCGCATCTTCTTG-3¡^−^
		R:5¡^−^-ACCGACCTTCACCATCTTGTCTA-3¡^−^
Alkaline phosphatase	ALP	F:5¡^−^-CCGATCGGGACTGGTACTC-3¡^−^
		R:5¡^−^-TCAGTTCTGTTCTTGGGGTACA-3¡^−^
Osteocalcin	OCN	F:5¡^−^-GAGGGCAGTAAGGTGGTGAA-3¡^−^
		R:5¡^−^-GTCCGCTAGCTCGTCACAAT-3¡^−^
Osteopontin	OPN	F:5¡^−^-TGTCCTCTGAAGAAACGGATG-3¡^−^
		R:5¡^−^-ACAGAATCCTCGCTCTCTGC-3¡^−^
Bone sialoprotein	BSP	F:5¡^−^-CGGCCACGCTACTTTCTTTA-3¡^−^
		R:5¡^−^-CCCTCCTCCTCCGAACTATC-3¡^−^
Collagen type I	COLI	F:5¡^−^-CATGTTCAGCTTTGTGGACCT-3¡^−^
		R:5¡^−^-GGTTTCCACGTCTCACCATT-3¡^−^
Runt-related transcription factor 2	RunX2	F:5¡^−^-CTTCACAAATCCTCCCCAAG-3¡^−^
		R:5¡^−^-GAGGCGGTCAGAGAACAAAC-3¡^−^
Osterix	OSX	F:5¡^−^-CACTGGCTCCTGGTTCTCTC-3¡^−^
		R:5¡^−^-GCTGTTGAGTCTCGCAGAGG-3¡^−^
Bone morphogenetic protein 2	BMP2	F:5¡^−^-AGACCACCGGCTGGAGAG-3¡^−^
		R:5¡^−^-TGAGAAACTCATCAGTAGGGACAG-3¡^−^
CCAAT/enhancer binding protein, alpha	C/EBPa	F:5¡^−^-AAGTCGGTGGATAAGAACAGC-3¡^−^
		R:5¡^−^-TCAACTCCAACACCTTCTGC-3¡^−^
Adipocyte lipid-binding protein	AP2	F:5¡^−^-GATTTCCTTCAAACTGGGCG-3¡^−^
		R:5¡^−^-TGACACATTCCACCACCAGC-3¡^−^
Lipoprotein lipase	LPL	F:5¡^−^-CAGCTGGGCCTAACTTTGAG-3¡^−^
		R:5¡^−^-AATGGCTTCTCCAATGTTGC-3¡^−^

### Microarray analysis

To compare the gene expression of cells in each group, microarray analysis was performed by Shanghai OE Biotech Co., Ltd. Simply, the total RNA was extracted according to the method mentioned above and its clean-up was performed using QIAGEN RNeasy Kit. The double strand cDNA was synthesized using 500 ng of total RNA. The *in vitro* transcription was performed to synthesize RNA amplification (aRNA). Samples were labeled using the GeneChip 3′IVT Express Kit (Affymetrix). The labeled aRNA was fragmented (35–200 nt) and hybridized to a GeneChip Rat Genome Array (Affymetrix). The size of aRNA fragmentation was checked by electrophoresis using the Agilent 2100 Bioanalyzer (Agilent Technologies). The hybridization was performed for 16 h at 60 rpm and 45 °C in the GeneChip Hybridization Oven 640 (Affymetrix). The Gene Chip Fluidics Station 450 (Affymetrix) was used to wash and stain the probe array according to the manufacturer’s protocols. The scanning of the samples was performed using the GeneChip Scanner 3000 (Affymetrix).

Affymetrix GeneChip Command Console (version 4.0, Affymetrix) was used to analyze array images to get raw data. Next, Genesrping software (version 12.5; Agilent Technologies) was employed to finish the basic analysis with the raw data. To begin with, the raw data was normalized with the MAS5 algorithm. The probes that at least 100.0 percent of samples in any 1 out of 2 conditions have flags in “P” were chosen for further data analysis. Differentially expressed genes were then identified through fold change. The threshold set for up- and down-regulated genes was a fold change ≥2.0.

### The osteogenic and adipogenic differentiation assay

To investigate the difference of cell pluripotency after 7 days expanding under the different culture conditions the cells were digested with 0.25% trypsin and transplanted into 6-well plate and cultured with osteogenic or adipogenic induction medium for 21 days respectively.

The osteogenic induction medium was consisted of L-DMEM supplemented with 10% FBS, 100 nmol/L dexamethasone, 10 mmol/L sodiumβ-glycerophosphate, and 0.05 mmol/L L-ascorbic acid 2-phosphate (Sigma) and replaced every 3 days. Von kossa staining and quantitative real-time PCR (qPCR) for osteoblastic markers were used for analysing the differences of the osteogenic ability among the 3 groups.

For adipogenic differentiation analysis, cells in each group were incubated in H-DMEM medium supplemented with 1 mmol/L dexamethasone (Sigma), 0.2 mmol/L indomethacin(Sigma), 10 mg/mL insulin(Roche), 0.5 mmol/L 3-isobutyl-1- methyl-xanthine (IBMX) (Sigma), and 10% FBS for 21 days. The adipogenic induction medium was replaced every 3 days. Oil red O staining and quantitative real-time PCR (qPCR) for adipogenic gene expression were used for analysing the differences of the adipogenic ability among the 3 groups.

### Oil red O staining

Each group sample was fixed in 4% formalin for 5 min. 0.5% Oil red O solution (sigma) was prepared in isopropanol and diluted 3:2 (v:v) with deionized water. Each sample was incubated with 1 mL Oil red O for 15 min at room temperature. After rinsed 3 times with PBS, samples were visualized under D5100 Digital Camera (Nikon).

### Von Kossa staining

The cells were washed twice with PBS and fixed in 4% paraformaldehyde for 30 min and then rinsed with deionized water. After a brief air dry, the samples were exposed to ultraviolet light in 1% aqueous silver nitrate under UV exposure for 30 min. Calcium deposition was appeared as black spots, and then the samples were rinsed fully with distilled water and 5% sodium thiosulfate to fix the positive dark staining and remove excess silver nitrate. Then the samples were visualized under D5100 Digital Camera (Nikon).

### Statistical analysis

All data were performed at least three times and expressed as the mean ± standard deviation (SD). Statistical analysis was performed with one-way ANOVA test and *p* < 0.05 was considered as significant.
